# Relationship between platelet parameters and sudden sensorineural hearing loss: a systematic review and meta-analysis

**DOI:** 10.1042/BSR20181183

**Published:** 2018-11-14

**Authors:** Shuaifei Ji, Xuemin Chen, Heng Shi, Babo Zhang, Shun Yao, Senlin Deng, Chunlong Tian, Jun Jiang, Fei Chen, Xiaocheng Wang

**Affiliations:** 1 School of Basic Medicine, Air Force Medical University, Xi’an, 710032, China; 2 Center of Clinical Aerospace Medicine, School of Aerospace Medicine, Key Laboratory of Aerospace Medicine of Ministry of Education, Air Force Medical University, Xi’an, 710032,China; 3 Department of ENT, Tianshui 407 Hospital, Tianshui, 741000, China; 4 People’s Liberation Army Troop 94587, Lianyungang, 222300, China

**Keywords:** mean platelet volume, meta-analysis, platelet distribution, platelet count width, Sudden sensorineural hearing loss

## Abstract

Recent years, the discussion about whether platelets participant in the development of sudden sensorineural hearing loss (SSHL) continues and many studies on the relationship between them have come to our attention. Some studies believe that platelet parameters have significantly changed in patients with SSHL, while some not, controversially. Therefore, to investigate the association between platelet parameters, including mean platelet volume (MPV), platelet distribution width (PDW) and platelet count (PLT), and SSHL, expecting to resolve controversy and provide clinical evidence for diagnosis and monitoring of SSHL. Basic methods: Literature was retrieved searching electronic databases (PubMed, Embase, Cochrane, and Scopus) and searching references of related articles by hand. A total of 18 case–control studies involving 1837/1734 subjects (SSHL/control) were included. Meta-analysis showed there was no difference between the patients who suffered SSHL and healthy controls in MPV level [standard mean difference (SMD) (95% confidence interval (CI)) = 0.16 (−0.07, 0.40), *I*^2^ = 80%, *P*<0.00001] and PLT [SMD (95% CI) = −0.03(−0.18, 0.12), *I*^2^ = 73%, *P*<0.00001]. While PDW exhibited significant difference [SMD (95% CI) = 0.85 (0.20, 1.49), *I*^2^ = 93%, *P*<0.00001]. Subgroup analysis about geographical area suggested PLT have obvious evidence for SSHL in Eastern country [SMD (95% CI) = 0.23 (0.14, 0.33), *I*^2^ = 0%, *P*=0.81]. Our study did not support a correlation between MPV and SSHL, while PLT may have clinical significance for SSHL in Eastern country. With insufficient data to explore the resource of heterogeneity for PDW, there is no decisive conclusion reached.

## Introduction

Sudden sensorineural hearing loss (SSHL) or sudden deafness, is typically defined as a hearing loss of at least 30 decibels occurring over at least three consecutive frequencies and lasting at least 3 days [1]. The estimated incidence varies from approximately 5 to 20 up to 160 per 100000 people per year [2]. The patient typically first notices symptoms upon awakening and describes an aural fullness/blockage. They may also experience tinnitus (usual), dizziness, or vertigo [1]. SSHL is considered to be a medical emergency, and, as such, requires prompt evaluation and treatment [3]. So far, the diagnosis and monitor of SSHL are mainly dependent on audio acuity, eardrum test and imaging, lacking of simple and quick serum markers. Recent years, many studies have showed that vascular events (vascular occlusions, hypostasis, ischemia, etc.) are assumed to be involved with the pathogenesis of SSHL [4–6]; therefore, some researchers focus on the clinical significance of platelet parameters (mean platelet volume (MPV), platelet distribution width (PDW), and platelet count (PLT)) in SSHL to explore whether platelet parameters can sever as markers to provide assistant for diagnosis and monitor of SSHL. However, it is a controversial topic whether platelet parameters are associated with SSHL, some hold positive attitude [7–9] and some are in the opposite [10–14]. Therefore, we carry out this systematic review and meta-analysis to expect to solve this controversy and provide clinical evidence for diagnosis and monitor of SSHL.

## Materials and methods

### Literature search and selection criteria

This systematic review and meta-analysis is reported in accordance with the Preferred Items for Systematic Reviews and Meta-analysis (PRISMA) Statement. Studies were retrieved by searching electronic databases (PubMed, Embase, Cochrane and Scopus) and searching references of related articles by hand. These computer searches were from the creation of the databases up to December 2017. Retrieval strategy of PubMed as follows: (((((Sudden Hearing Loss or Deafness, Sudden or Sudden Deafness))) OR “Hearing Loss, Sudden” [Mesh])) AND (((((platelet count) OR platelet)) OR ((platelet distribution width) OR PDW)) OR ((“Mean Platelet Volume” [Mesh]) OR mean platelet volume)).

The inclusion criteria were as follows: (1) Participants: SSHL patients without other illness related to platelet activity, such as venous thrombosis, cardiovascular disease; (2) Exposures: platelet parameters level; (3) Comparatives: healthy controls; (4) Type of studies: independent case–controlled studies using either a hospital-based or population-based design; (5) Publications with English or Chinese, the latter must be on Medline.

Exclusion criteria included the following: (1) Duplicated data; (2) Case reports and studies where the original data could not be extracted; (3) Animal experiments and basic research; (4) Reviews and letters.

### Data extraction

Two authors (S.F. Ji and X.M. Chen) independently extracted the original data. Disagreement was resolved by discussion. If the two authors could not reach a consensus, the result was reviewed by a third author (H. Shi). The extracted data consisted of the following items: the first author’s name, publication year, population (ethnicity), methods, study design, matching criteria, sex, total number of cases and controls, and age (years).

### Quality assessment

The Newcastle-Ottawa Scale (NOS) was used to assess the methodological quality of the individual studies. Each study was evaluated and scored based on three criteria: selection (4 stars), comparability (2 stars), and exposure (3 stars). The NOS point scale ranged from 0 to 9 stars, the researches with NOS ≥7 stars were considered high quality.

### Statistical analysis

We utilized Review manager 5.3 and Stata 14.0 software to perform the meta-analysis in the present study. Heterogeneity among studies was assessed by the *I*^2^ statistic, and *P*<0.10 and *I*^2^ > 50% indicated evidence of heterogeneity. If heterogeneity existed among the studies, a random-effects model was used to estimate the pooled standard mean difference (SMD). Otherwise, a fixed-effects model was adopted. The SMD and corresponding 95% confidence interval (CI) were utilized to assess the associations. Subgroups analysis regarding geographical area, median of detection of patients, sample size, with EDTA anticoagulation treatment and NOS quality, were also performed to explore source of heterogeneity. Egger’s and Begg’s tests (*P*<0.05) can demonstrate a statistically significant publication bias and were conducted with the Stata 14.0 software, if there was any publication bias, trim, and fill method was implemented. Sensitive analysis was also conducted by changing effect model.

## Results

### Selection and characteristics of studies

**Figure 1:**
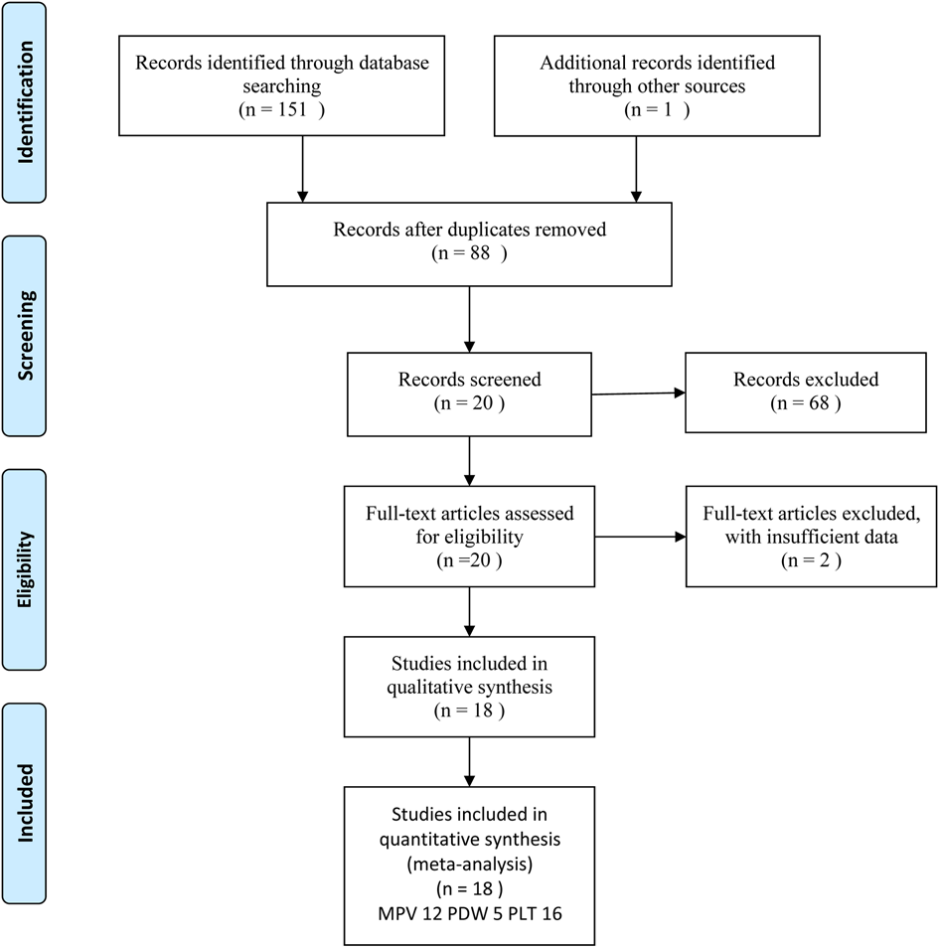
Flow diagram for literature selection

We retrieved a total of 152 studies in the literature search (Figure 1). After duplicates were removed, only 88 full-text studies remained. Then, 70 articles, with 45 no-related reports, 17 reviews and letters, 2 animals research, 4 case reports and 2 insufficient-data studies, were excluded after screening abstracts and full-texts. Finally, 18 case–control studies were included [7–24], 12 studies mentioned MPV [7–14,16–18,24], 5 studies detected PDW [7,8,12,16,17], and 16 studies reported PLT level [7–10,12,13,15–24]. And 11 studies were performed in Turkey [7,8,10–13,15,17–19,23], 3 in China [20,21,24], 2 in Korea [9,23], 1 in the Czech Republic [14], and 1 in Iran [16]. Details about NOS assessment and PRISMA are in Supplementary S1 and Supplementary S2. The characteristics of the eligible studies are shown in Tables 1 and 2.

**Table 1:** Main characteristics of eligible studies

First author (location, year)	Group	Subjects (*N*)	Age (years)	Male/Female	NOS
Durmus (Turkey, 2016) [8]	Case	140	47.65 ± 16.14	88/52	7
	Control	132	44.42 ± 16.22	32/100	
Mirvakili (Iran, 2016) [16]	Case	108	45.15 ± 14.42	61/47	8
	Control	108	43.15 ± 11.54	61/47	
Ozturk (Turkey, 2014) [12]	Case	39	39.1 ± 12.8	23/16	6
	Control	40	38.9 ± 11.2	19/21	
Ulu (Turkey, 2013) [7]	Case	40	44.6 ± 16.4	20/20	8
	Control	40	46.8 ± 9.5	23/17	
Sagit (Turkey, 2012) [17]	Case	31	37.45 ± 15.70	17/14	8
	Control	31	35.77 ± 14.93	16/15	
Lee (Korea, 2017) [9]	Case	46	14.70 ± 2.81	26/20	8
	Control	46	15.20 ± 2.28	30/16	
Kum (Turkey, 2015) [13]	Case	59	46.10 ± 11.91	38/21	8
	Control	59	42.84 ± 11.85	31/28	
Sun (China, 2017) [24]	Case	129	43.77 ± 14.44	68/61	8
	Control	31	51.06 ± 10.01	16/15	
Ezerarslan (Turkey, 2016) [18]	Case	62	51 ± 19	26/36	7
	Control	49	49 ± 16.2	16/33	
Karli (Turkey, 2013) [10]	Case	46	45.39 ± 15.70	25/21	8
	Control	46	41.38 ± 16.70	24/22	
Koçak (Turkey, 2016) [11]	Case	93	32.3 ± 7.9	41/52	8
	Control	93	31.4 ± 8.1	46/47	
Bláha (Czech Republic, 2014) [14]	Case	54	54.2 ± 14.9	32/22	6
	Control	38	32.6 ± 7.4	19/19	
Bulğurcu (Turkey, 2017) [23]	Case	21	13.7 ± 3.2	13/8	6
	Control	24	14.8 ± 2.9	12/12	
İkincioğullar (Turkey, 2014) [19]	Case	102	48.94 ± 13.86	54/48	8
	Control	119	47 ± 9.63	65/54	
Koçak (Turkey, 2017) [15]	Case	45	31.1 ± 7.4	25/20	8
	Control	47	32.4 ± 8.1	19/28	
Seo (Korea, 2014) [22]	Case	348	48.19 ± 15.22	171/177	7
	Control	537	48.22 ± 11.6	288/249	
Bao (China, 2015) [21]	Case	424	44.22 ± 14.92	216/208	5
	Control	244	42.35 ± 14.71	132/112	
Lu (China, 2008) [20]	Case	50	14–69*	32/18	5
	Control	50	14–69*	32/18	

* Range.

**Table 2:** Main characteristics of eligible studies

Study	MPV (fl)			PDW (fl)			PLT (10^3^/μl)		
	Case	Control	*P*	Case	Control	*P*	Case	Control	*P*
Durrmus	8.98 ± 1.73	9.12 ± 0.84	&	17.39 ± 6.27	10.06 ± 1.99	#	228.33 ± 65.21	258.59 ± 50.63	#
Mirvakili	10.02 ± 0.76	9.85 ± 0.67	&	12.45 ± 1.50	12.11 ± 1.24	&	228.51 ± 62.45	222.86 ± 36.80	&
Ozturk	8.19 ± 1.07	8.01 ± 1.05	&	17.7 ± 0.89	17.6 ± 1.04	&	257 ± 57	268 ± 63	&
Ulu	10.5 ± 0.9	9.6 ± 0.5	#	13.4 ± 2.1	11.1 ± 1.0	#	232.9 ± 59.8	276.5 ± 62.1	#
Sagit	9.01 ± 1.24	8.21 ± 0.76	#	16.29 ± 1.10	14.65 ± 2.13	#	258.03 ± 58.28	249.06 ± 61.96	&
Lee	7.89 ± 0.92	8.27 ± 0.74	#				287.52 ± 59.36	286.22 ± 55.99	&
Kum	9.83 ± 1.50	9.98 ± 0.07	&				249.44 ± 48.16	244.86 ± 47.25	&
Sun	10.47 ± 1.43	9.75 ± 1.66	#				217.46 ± 53.67	202.48 ± 46. 61	&
Ezerarslan	8.1 ± 1.2	8.4 ± 1.2	&				234 ± 53.5	236 ± 39	&
Karli	8.25 ± 0.86	7.98 ± 0.87	&				243 ± 81.5	275 ± 82.5	&
Koçak^1^	8.2 ± 2.2	8.7 ± 1.3	&						
Bláha	10.68 ± 1.1	10.47 ± 0.8	&						
Bulğurcu							247.12 ± 53.23	262.11 ± 41.18	&
İkincioğullar							263.27 ± 64.11	259.32 ± 64.80	&
Koçak^2^							257 ± 61	257 ± 47	&
Seo							252.40 ± 60.07	238.64 ± 49.98	#
Bao							240.47 ± 41.75	229.51 ± 48.88	#
Lu							210.72 ± 49.40	205.52 ± 47.78	&

Koçak^1^, (Turkey, 2016); Koçak^2^, (Turkey, 2017). ^#^*P*<0.05, ^&^*P*≥0.05.

## Meta-analysis

### Differences of MPV between SSHL patients and controls

**Figure 2:**
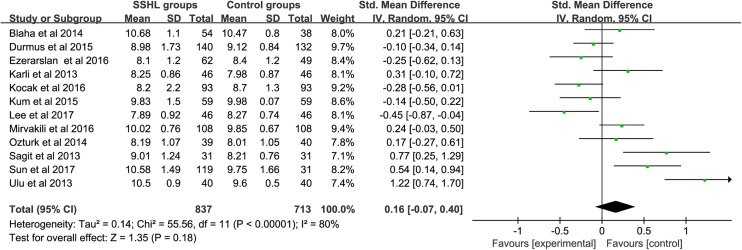
Difference of MPV between SSHL patients and controls

**Figure 3:**
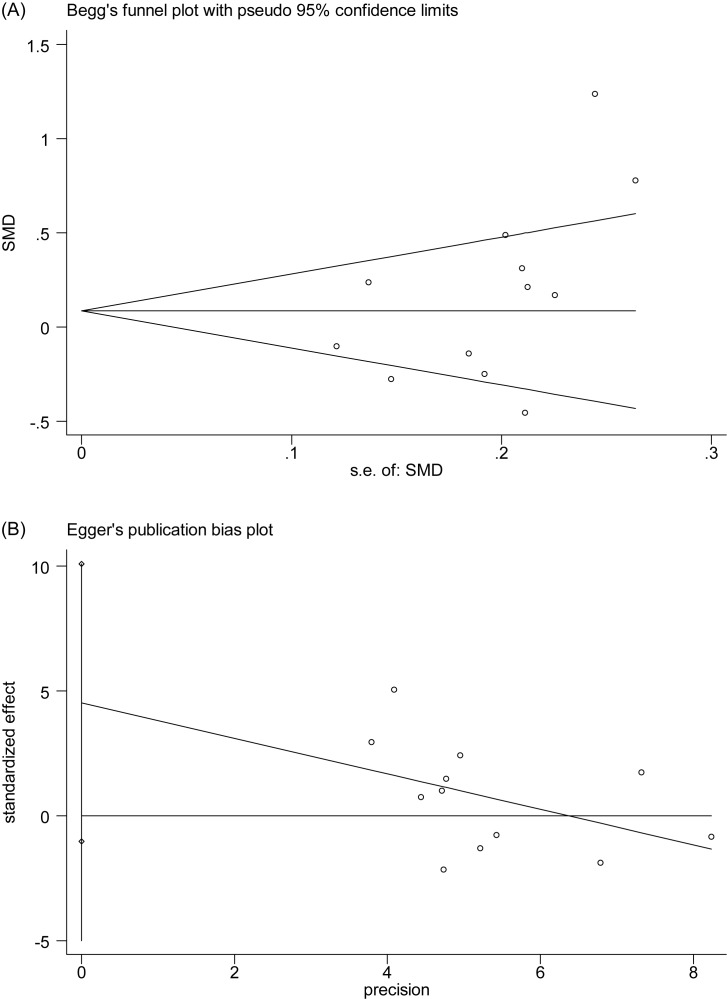
Begg’s test (**A**) and Egger’s test (**B**) for publication bias assessment of MPV.

There were 12 studies reporting the association between MPV level and SSHL compared with control groups (Figure 2). An *I*^2^ test indicated that the heterogeneity was significant (*I*^2^ = 80%, *P*<0.00001), therefore, a random-effects model was applied to perform the meta-analysis. The results showed that MPV level in the SSHL group was not different from that in the control group [SMD (95% CI) = 0.16 (−0.07, 0.40)]. The weights were evenly distributed, and sensitive analysis showed that re-synthesized result was similar to previous one after fixed-effects model conducted [SMD (95% CI) = 0.09 (−0.02, 0.19)], which suggested the result is reliable. In addition, Egger’s test (*P*=0.097) and Begg’s test (*P*=0.193) showed that there was no publication bias (Figure 3). Subgroup analysis (Table 3) by geographical area demonstrated that there were no significant difference both in Eastern countries [SMD (95% CI) = 0.05(−0.93,1.02)] and Western countries [SMD (95% CI) = 0.18 (−0.07,0.43)], and the similar results appeared in sample size ≥100 [SMD (95% CI) = 0.20 (−0.15, 0.54)] and <100 [SMD (95% CI) = 0.15 (−0.17,0.48)], NOS ≥7 [SMD (95% CI) = 0.16 (−0.12, 0.44)] and <7 [SMD (95% CI) = 0.19 (−0.11,0.49)], and EDTA with mentioned [SMD (95% CI) = 0.42 (−0.00, 0.85)] and no-mentioned [SMD (95% CI) = −0.02 (−0.28, 0.24)]. Result of sensitive analysis showed there did not appear significant change [SMD (95% CI) = 0.09 (−0.02, 0.19)].

**Table 3:** Summary effects of subgroup analysis results

Subgroup	*N*^#^	SMD (95% CI)	*P*-value	Heterogeneity	
				*P-*value	*I*^2^ (%)
**MPV**	837/713	0.16 (–0.07, 0.40)	=0.18	<0.00001	80
Geographical area					
Eastern country	165/77	0.05 (–0.93,1.02)	=0.93	=0.0007	91
Western country	672/636	0.18 (–0.07, 0.43)	=0.15	<0.00001	80
Sample size of patients					
Size ≥100	367/271	0.20 (–0.15, 0.54)	=0.26	=0.02	76
Size <100	470/442	0.15 (–0.17,0.48)	=0.35	<0.00001	83
EDTA					
Mentioned	358/351	0.42 (–0.00, 0.85)	=0.05	<0.0001	86
No-mentioned	479/362	–0.02 (–0.28, 0.24)	=0.90	=0.003	70
NOS					
≥7	744/635	0.16 (–0.12, 0.44)	=0.25	<0.0001	84
<7	93/78	0.19 (–0.11, 0.49)	=0.22	=0.89	0
**PLT**	1690/1603	–0.03 (–0.18, 0.12)	=0.72	<0.00001	73
Geographical area					
Eastern country	997/908	0.23 (0.14, 0.33)	<0.00001	=0.81	0
Western country	693/695	–0.15 (–0.33,0.03)	=0.11	=0.003	62
Sample size of patients					
Size ≥100	1251/1171	0.07(–0.16, 0.30)	=0.54	<0.00001	85
Size <100	439/432	–0.11(–0.27, 0.06)	=0.20	=0.15	32
EDTA					
Mentioned	853/669	–0.12(–0.40, 0.15)	=0.38	<0.00001	82
No-mentioned	837/934	0.08(–0.06, 0.22)	=0.27	=0.12	38
NOS					
≥7	1177/1269	–0.06(–0.24, 0.12)	=0.52	<0.00001	75
<7	513/334	0.13(–0.10, 0.36)	=0.28	=0.19	41

*N*^#^, case/control.

### Difference of PDW between SSHL patients and controls

**Figure 4:**
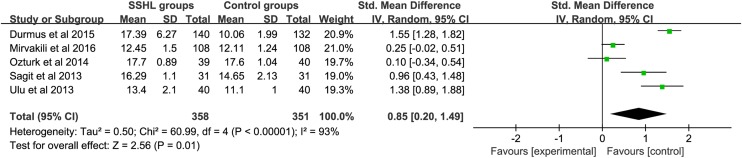
Differences of PDW between SSHL patients and controls

**Figure 5:**
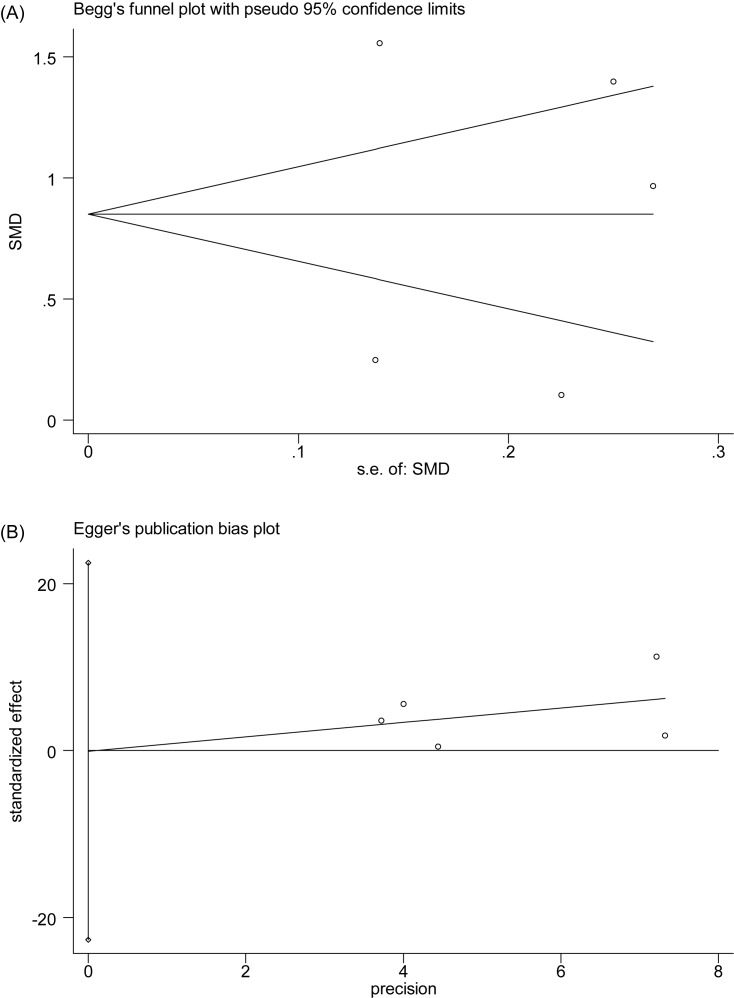
Begg’s test (**A**) and Egger’s test (**B**) for publication bias assessment of PDW.

There were five studies reporting the association between PDW level and SSHL compared with control groups (Figure 4). An *I*^2^ test indicated that the heterogeneity was significant (*P*<0.00001, *I*^2^ = 93%); therefore, a random-effects model was applied to perform the meta-analysis. The results showed that there was statistical significance in the comparison of SSHL and control group [SMD (95% CI) = 0.85 (0.20, 1.49)]. The weights were evenly distributed, and sensitive analysis showed that re-synthesized result did not change obviously after fixed-effects model conducted [SMD (95% CI) = 0.85 (0.69, 1.00)], which suggested it is reliable. Likewise, there was no significant publication bias according to Egger’s test (*P*=0.993) and Begg’s test (*P*=0.806) (Figure 5). With insufficient data, we did not conduct subgroup analysis.

### Difference of PLT between SSHL patients and controls

**Figure 6:**
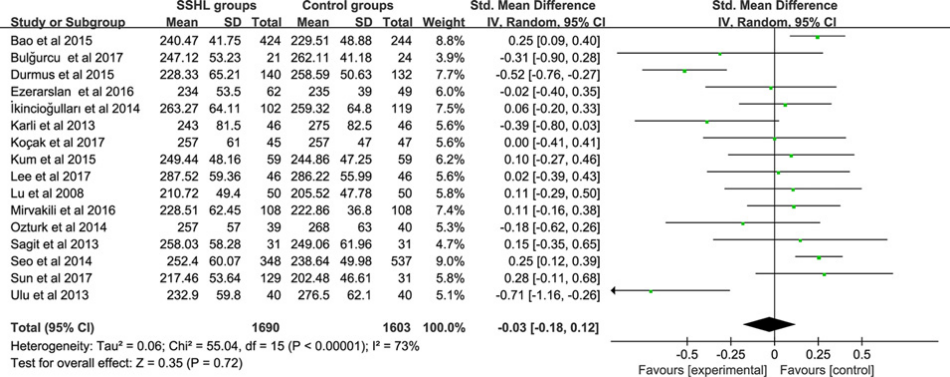
Differences of PLT between SSHL patients and controls

**Figure 7:**
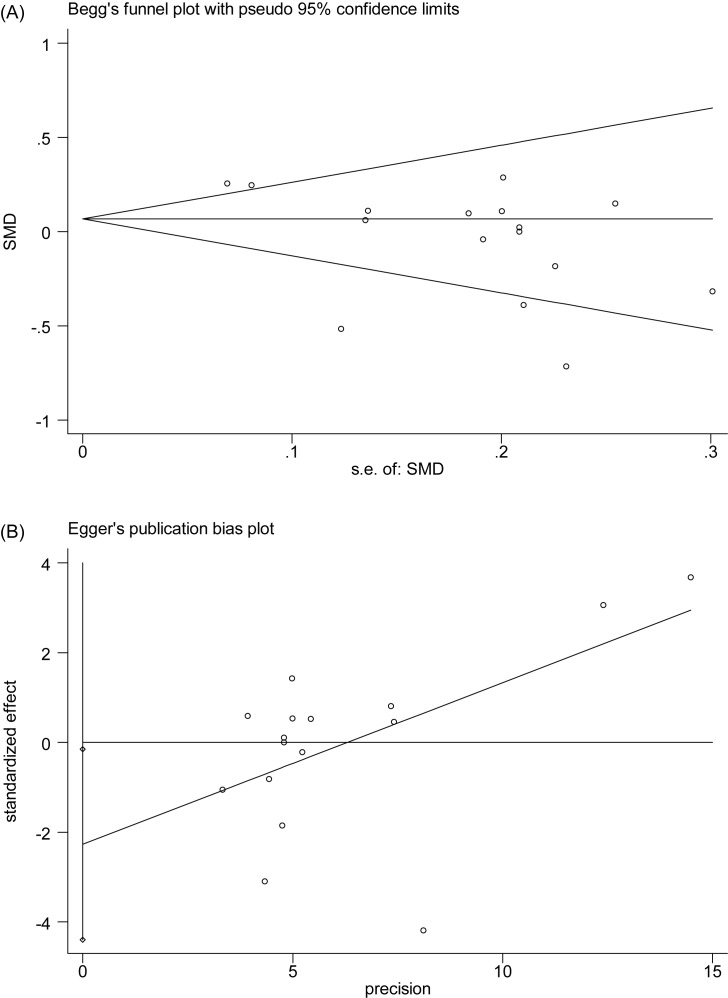
Begg’s test (A) and Egger’s test (B) for publication bias assessment of PLT.

**Figure 8:**
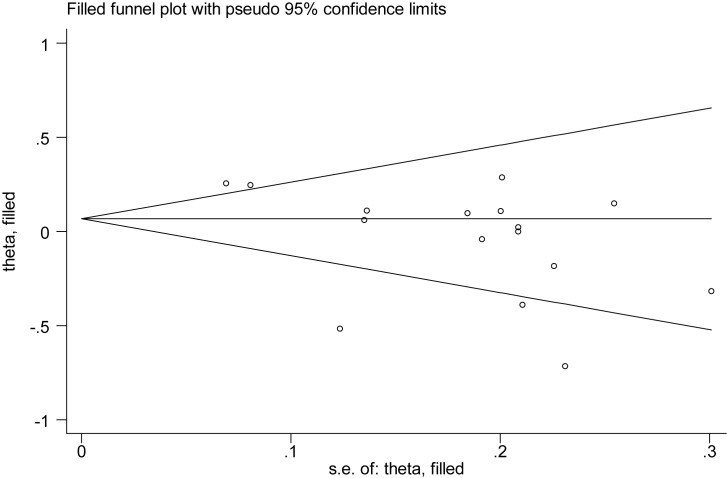
Filled funnel plot for publication bias assessment of PLT

There were 15 studies reporting the association between PLT level and SSHL compared with control groups (Figure 6). An *I*^2^ test indicated that the heterogeneity was significant (*P*<0.00001, *I*^2^ = 73%); therefore, a random-effects model was applied to perform the meta-analysis. The results showed that there was no statistical significance in the comparison of SSHL and control group [SMD (95% CI) = −0.03 (−0.18, 0.12)]. Likewise, sensitive analysis with fixed-model [SMD (95% CI) = 0.07 (−0.00 0.14)] showed our result was reliable. Although there was publication bias according to Egger’s test (*P*=0.037) and Begg’s test (*P*=0.043) (Figure 7), the trim and fill test suggested it did not have an impact on our result (Figure 8). Similar to MPV, subgroup analysis (Table 3) showed that there were no significant difference in sample size ≥100 [SMD (95% CI) = 0.07 (−0.16,0.30)] and <100 [SMD (95% CI) = −0.11 (−0.27, 0.06)], NOS ≥7 [SMD (95% CI) = −0.06 (−0.24,0.12)] and <7 [SMD (95% CI) = 0.13 (−0.10,0.36)], and EDTA with mentioned [SMD (95% CI) = −0.12 (−0.40,0.15)] and no-mentioned [SMD (95% CI) = 0.08 (−0.06,0.22)]. But in respect of geographical area, there was significant result in Eastern country [SMD (95% CI) = 0.23 (0.14, 0.33)], but not in Western country [SMD (95% CI) = −0.15 (−0.33, 0.03)].

## Discussion

Although platelet parameters, such as MPV, PDW, and PLT, have exhibited diagnostic significance in various diseases and conditions [25,26], it is unclear that whether there exists clinic significance of them in SSHL. This study is the first meta-analysis of published studies to explore the relationship between platelet parameters and SSHL. Our results did not support there was relationship between MPV and SSHL, but PLT may be applied to clinical practice of Eastern country SSHL patients.

SSHL is a clinical condition characterized by the acute onset of unilateral or bilateral hearing loss greater than 30 dB over three consecutive frequencies in less than 72 h. Different theories about the pathogenesis of SSHL have been proposed, including vascular incidents, vascular occlusions, viral, bacterial, and protozoan infections, intracochlear membrane rupture, autoimmune disorders, and side effects from ototoxic medications [27]. There are a series of studies that prove that the disturbance of blood circulation plays a large role in SSHL [28]. As the cochlear artery is a terminal branch of the anterior inferior cerebellar artery, with a small inner diameter and insignificant collateral circulation, any pathology in this location that influences collateral blood supply may damage the cochlea and cause acute hearing loss [10].

Platelets are the smallest cells of the peripheral blood, and they are involved in hemostasis and the formation of thrombosis in blood vessels. Mediators and substances released by platelets play a significant role in the progress of coagulation, inflammation, thrombosis, and atherosclerosis. MPV is a commonly used parameter to indicate the volume, function, and activity of platelets. It has been proven that MPV levels increase in vascular events such as atherosclerosis, acute syndromes, venous and arterial thrombosis, or thromboembolism [29]. The increased MPV contributes to the prethrombotic state in acute syndromes. PLT and PDW are other important platelet parameters. PLT refers to the number of platelets in per unit volume of blood. PDW reflects the variation in size of platelets in a blood sample.

In recent years, a series of studies concerning the relationship between platelet parameters and the occurrence and severity of SSHL have been performed, but there was no consistent conclusion. Ulu et al. [7] and Durmus et al. [8] observed significantly higher MPV, PDW, and PLT in SSHL patients than controls and concluded that they could be taken into consideration as potential novel markers in the assessment of SSHL. By contrast, Lee et al. [9] reported the relationship between MPV and SSHL in children and found MPV was significantly decreased in the SSHL group, which might reflect that the mechanisms of pediatric SSHL are different from those of adults. We did not further analyze the differences between children and adults due to the limitation of fewer studies about the former. Furthermore, other studies (10–16) have shown no statistical correlation between platelet parameters and SSHL. It is unusual to find that there seems to be a paradox, which limits our interpretation of these experimental results.

MPV, PDW, and PLT are susceptible to many vascular risk factors including age, smoking, diabetes mellitus, hypertension, hyperlipidemia, and obesity [30]. It is advisable for researchers to rule out patients with active inflammations, suspicion of an autoimmune inner ear disease, known etiology of SSHL, history of otologic surgery or head and neck trauma, previously diagnosed cardiovascular disease, chronic diseases such as diabetes mellitus, hypertension, hyperlipidemia, obesity, antiaggregant or anticoagulant drug use, chronic alcohol consumption, and smoking. These factors may influence the outcome of MPV, PDW, and PLT assessments. Although there are a number of exclusion criteria in the studies to standardize the results, MPV, PDW, and PLT may still be affected by many other minor factors, such as nasal septum deviations, major depression, insulin resistance, and erectile dysfunction [17,31–33], which were not taken into consideration and thus not excluded. Our aim with the exclusion criteria was to show the possible correlation with SSHL and platelet parameters but, in daily practice, comorbidities are frequently observed in patients with SSHL. It is impossible for us to adjust for all the variables between SSHL and control groups.

In the methods section, in order to measure MPV, venous blood samples were collected into tubes containing EDTA. However, in an earlier study, Bath et al. [34] found that measurements in EDTA can be unreliable since MPV increases significantly in a time-dependent manner. One can readily measure MPV by clinical hematology analyzers using sodium citrate as the anticoagulant. Therefore, we performed a pooled analysis about EDTA, although only five studies [7,8,12,16,21] reported measuring MPV with EDTA. The analysis showed that the results did not change [SMD (95% CI) = -0.12(-0.40, 0.15)], which suggested that EDTA did not influence final results.

In respect of MPV in SSHL, pooled results did not support there exit the association, but quality of included studies may be main resource of heterogeneity. Although we discover PDW exhibit difference between SSHL group and control group, there was no enough data to explore resources of heterogeneity, in addition to considering small studies included, we could not reach a conclusion impetuously. A total of 16 studies included to study the relationship between PLT and SSHL, our result also did not uphold the relationship completely. However, subgroup analysis by geographical area showed that there was significant difference in Eastern country, while other subgroups not, which suggested geographical area may be the source of heterogeneity. Sensitive analysis revealed that our results were stable. And there was no publication bias, if any, it did not impact our results. All of them demonstrated our results were reliable.

There were several limitations exiting in this analysis need to be carefully considered. First, all the including researches were case–control studies, and we could not ensure other factors matched completely. Second, in terms of PDW, too few articles included to find the source of heterogeneity, so we could not reach a conclusion. Third, although we have implemented full subgroups analysis, we failed to discover the other sources heterogeneity about MPV and PLT. Finally, with insufficient data, we did not explore the threshold.

In summary, we concluded that MPV failed to apply to diagnosis and monitor of SSHL, while PLT might be a positive factor for SSHL in Eastern country. The clinical significance of PDW for SSHL is remaining to be confirmed with more well-designed and large-scale studies.

## Supporting information

**Supplementary 1:** NOS scores of included studies.

## Abbreviations

CI: confidence interval
MPV: mean platelet volume
NOS: Newcastle-Ottawa Scale
PLT: platelet count
PDW: platelet distribution width
PRISMA: Preferred Items for Systematic Reviews and Meta-analysis
SMD: standard mean difference
SSHL: sudden sensorineural hearing loss
EDTA: ethylenediaminetetraacetic acid
